# Prognosis of breast cancer is associated with one-carbon metabolism related nutrients among Korean women

**DOI:** 10.1186/1475-2891-11-59

**Published:** 2012-08-28

**Authors:** Yunhee Lee, Sang-Ah Lee, Ji-Yeob Choi, Minkyo Song, Hyuna Sung, Sujee Jeon, Sue K Park, Keun-Young Yoo, Dong-Young Noh, Sei-Hyun Ahn, Daehee Kang

**Affiliations:** 1 Department of Biomedical Sciences, Seoul National University, Seoul, Republic of Korea; 2 Department of Preventive Medicine, Kangwon National University School of Medicine, Kangwon, Republic of Korea; 3 Department of Molecular Medicine and Biopharmaceutical Sciences, Graduate School of Convergence Science and Technology and College of Medicine or College of Pharmacy, Seoul, Republic of Korea; 4 Department of Preventive Medicine, Seoul National University, Seoul, Republic of Korea; 5 Department of Surgery, Seoul National University College of Medicine, Seoul, Republic of Korea; 6 Department of Surgery, University of Ulsan College of Medicine and Asan Medical Center, Ulsan, Republic of Korea; 7 Department of Preventive Medicine, Seoul National University College of Medicine, 103 Yongon (Daehangno), Jongno-gu, Seoul, Republic of Korea

**Keywords:** Breast cancer prognosis, One-carbon metabolism, Vitamin B_2_, Vitamin B_6_, Folate

## Abstract

**Background:**

The 5-year survival rate for breast cancer among Korean women has increased steadily; however, breast cancer remains the leading cause of cancer mortality among women. One-carbon metabolism, which requires an adequate supply of methyl group donors and B vitamins, may affect the prognosis of breast cancer. This aim of this study was to investigate the associations of dietary intake of vitamin B_2_, vitamin B_6_ and folate before diagnosis on the prognosis of breast cancer.

**Methods:**

We assessed the dietary intake using a food frequency questionnaire with 980 women who were newly diagnosed and histopathologically confirmed to have primary breast cancer from hospitals in Korea, and 141 disease progression events occurred. Cox’s proportional hazard regression models were used to estimate the hazard ratio (HR) and 95% confidence interval (95% CI) adjusting for age, education, recruitment sites, TNM stage, hormone status, nuclear grade and total calorie.

**Results:**

There was no significant association between any one-carbon metabolism related nutrients (vitamin B_2_, B_6_ and folate) and the progression of breast cancer overall. However, one-carbon metabolism related nutrients were associated with disease progression in breast cancer patients stratified by subtypes. In ER + and/or PR + breast cancers, no association was observed; however, in ER–/PR– breast cancers, a high intake of vitamin B_2_ and folate statistically elevated the HR of breast cancer progression (HR = 2.28; 95% CI, 1.20-4.35, HR = 1.84; 95% CI, 1.02-3.32, respectively) compared to a low intake. This positive association between the ER/PR status and progression of the disease was profound when the nutrient intakes were categorized in a combined score (P_interaction_ = 0.018). In ER–/PR– breast cancers, high combined scores were associated with a significantly poor DFS compared to those belonging to the low score group (HR = 3.84; 95% CI, 1.70-8.71).

**Conclusions:**

In conclusion, our results suggest that one-carbon related nutrients have a role in the prognosis of breast cancer depending on the ER/PR status.

## Introduction

Breast cancer is the second most common malignancy in Korea. The 5-year survival rate for breast cancer among Korean women has increased from 78.0% during 1993–1995 to 89.5% during 2003–2007. Despite such marked improvement, breast cancer remains the second leading cause of cancer mortality among women in Korea [1]. Analyzing prognostic factors associated with breast cancer survival and recurrence is important for early detection and chemotherapy.

Known prognostic indicators such as pathological criteria including tumor size, lymph-node status and hormone receptor status are pathologically used in clinical practice [2]. Although numerous studies have shown that disease-free survival after breast cancer treatment may be partially predicted by pathological factors [3,4], they are not yet predicted prognostic factors for each patient in clinical practice.

Epigenetic aberrations occur in tumor cells; especially, CpG-island hypermethylation and global genomic hypomethylation are common features of breast cancer cells [5]. A lack of methyl supply can induce DNA global hypomethylation as well as incomplete conversion of dUMP to dTMP leading to uracil misincorporation into DNA [6]. One-carbon metabolism is a network of interrelated biochemical reactions that include the transfer of one-carbon groups [7], which have two major functions: DNA methylation and DNA synthesis [8].

The principal element of one-carbon metabolism is folate, since the one-carbon transfer reactions involve interconversion between several forms of this nutrient. Other important nutrients in one-carbon metabolism include vitamins B_2_, B_6_ and B_12_, which act as essential cofactors for one or more enzymes that catalyze one-carbon transfer reactions. Vitamin B_2_ is a cofactor for methylenetetrahydrofolate reductase, the critical folate-dependent enzyme. Vitamin B_6_ has a role in the conversion of tetrahydrofolate to 5, 10-methylenetetrahydrofolte, which is required for the synthesis of thymidylate and a precursor for purine synthesis [9,10].

Previous studies have suggested that a high status of one-carbon metabolism factors has reduced the risk of several cancers, including colorectal, pancreatic, esophageal, renal cell, and breast cancer [11-15]. In studies related to breast cancer risk, folate has been investigated many times. Although a number of cohort and case–control studies have suggested a protective effect for a high folate status on breast cancer risk, these results are far from conclusive. And other B vitamins did not showed any associations with breast cancer risk [15]. For studies on one-carbon metabolism related nutrients and breast cancer survival, a few investigated the association between folate intake and breast cancer survival with inconsistent results. In a Swedish mammography study, high folate intake before breast cancer diagnosis improved the prognosis of the breast cancer and overall survival [16]. However, in the Long Island Breast Cancer Study Projects and Nurses’ Health study, the association between vitamin B_2_, B_6_ and folate and breast cancer prognosis had null results [17,18].

One-carbon metabolism, which requires an adequate supply of methyl group donors and B vitamins, may modify the methylation profile of the genome, thus influencing breast cancer prognosis. The aim of this study was to investigate the associations of dietary intake of vitamin B_2_, vitamin B_6_ and folate before diagnosis on the prognosis of breast cancer.

## Materials and methods

### Study population

A total of 1,586 newly diagnosed breast cancer cases were recruited from Seoul National University Hospital and Asan Medical Center in Korea from 2004 to 2007 [19]. Before any adjuvant chemotherapy and/or surgery, baseline data were collected using questionnaires. Patients with a prehistory of cancer, multiple cancers at diagnosis, distant organ metastasis at diagnosis, *in situ* breast cancer and unobtainable of dietary assessment were excluded. Subjects with a total energy intake from 500 to 3,500 kcal/day were included for the final analysis. In the final analysis, a total of 980 invasive ductal carcinoma cancer patients diagnosed with stage I - III who underwent curative resection were included.

The study was approved by the Committee on Human Research of Seoul National University Hospital (IRB No. H-0503-144-004). All participants provided informed consent before their participation in the study.

### Data collection

The demographic characteristics of the participants were obtained by trained interviewers using a structured questionnaire. Information on demographic characteristics including age, education, reproductive and menstrual factors and lifestyle habits including smoking status, and alcohol consumption were collected.

A retrospective chart review was used to collect clinicopathological information including cancer stage, tumor size, lymph-node status, distant organ metastasis, estrogen receptor (ER) and progesterone receptor (PR) status, nuclear grade, surgical treatment and medical adjuvant therapy. Death was ascertained from Statistics Korea.

The food frequency questionnaire (FFQ) included 103 food items and detailed information on the FFQ has been described in a previous study [20]. During the in-person interviews, each participant was asked about their dietary habits for one year before the date of diagnosis. The frequency of servings was classified into nine categories: never or seldom, once a month, 2–3 times a month, one to two times a week, three to four times a week, five to six times a week, once a day, twice a day or three times or more every day. For food items with different seasonal availability, we requested that participants choose one category for how long they have eaten a particular food item from among four categories: 3, 6, 9 or 12 months. The portion size of each food item was classified as follows: small, medium or large. To help in understanding portion sizes, we provided pictures on serving sizes for food items on their corresponding pages. The reproducibility and validity of the FFQ were analyzed for 124 subjects in a previous study [20]. The FFQ was assessed twice at 1-year intervals for the reproducibility analysis. The correlation coefficients of the nutrients were 0.54, 0.44 and 0.43 for vitamin B_2_ (unpublished data), vitamin B_6_ and folate, respectively. FFQs were compared with 12-day diet records (3 days during each of the four seasons) for the validity analysis. The correlation coefficients of the nutrients were ranged from 0.15 to 0.31 for vitamin B_2_ (unpublished data), vitamin B_6_ and folate [20].

### Statistical analysis

Disease free survival (DFS) was defined as the time from the date of surgery to the date of the first locoregional recurrence, first distant metastasis, 2^nd^ primary cancer or death from any cause. Patients known to be alive with no evidence of disease were censored at the last follow-up date.

Cox’s proportional hazard regression models were used to estimate the hazard ratio (HR) and 95% confidence interval (95% CI). Multivariate Model 1 included age, TNM stage, hormone status and nuclear grade while Model 2 included age, education, recruitment sites, TNM stage, hormone status and nuclear grade. In addition, one-carbon metabolism related nutrients were adjusted for total calories in both Model 1 and Model 2. The adjusted variables in Model 1 were known factors of breast cancer prognosis and the variables added to Model 2 were known factors related to the intake of nutrients. Other covariates including tumor size and lymph node status were considered but not included in the final model, since the TNM stage was adjustment variable in the final model.

We performed analyses with vitamin B_2_, vitamin B_6_ and folate as continuous variables and as categorical variables in quartiles. To further assess the combined effects of the intake of one-carbon metabolism related nutrients including vitamin B_2_, vitamin B_6_ and folate on breast cancer survival, each vitamin was coded as 0 or 1 by median, and calculated using the sum of the numbers. The combined score was categorized into three groups which were low (score, 0), medium (score, 1–2) and high (score, 3).

Wald *P* values for trends were computed by treating categorical variables as ordinal variables. *P* for interaction was analyzed by multiplying two variables.

Additionally, stratified analyses were done according to age (≤39, 40–49, 50–59, and ≥60), menopausal status, BMI group (<25 kg/m^2^ and ≥25 kg/m^2^), alcohol consumption (<1/month, 1+/month), TNM stage (I-II and III), lymph-node status (negative and positive), hormone status (ER + and/or PR + and ER–/PR–) and nuclear grade (I-II and III).

All statistical analyses were done using SAS statistical software version 9.2 (SAS Institute, Cary, NC).

## Results

The median follow-up time was 5.3 years (range, 0.2-7.0 years). There were 141 DFS events including 77 deaths from any cause among all 980 patients. Table 1 summarizes the association between demographic and clinicopathological factors and progression in breast cancer patients. In this study, breast cancer patients who were estrogen receptor negative (ER–) and progesterone receptor negative (PR–) were 39.2% and 44.2%, respectively. Among demographic factors, patients with an education higher than the college level were associated with progression of breast cancer compared to the patients with an education lower than middle school. Among clinical factors, TNM stage, tumor size, lymph node status and hormone status were significant prognostic factors for breast cancer (*P* < 0.05).

**Table 1:** Hazard ratios for disease progression in breast cancer patients

	**All (N = 980)**	**Events (N = 141)**	**Model 1**^**a**^	**Model 2**^**b**^
Age (mean(SD))	48 (9.8)	49 (11.7)	1.02 (1.00-1.04)	1.01 (0.99-1.03)
≤39	191 (19.5)	30 (21.3)	1.00	1.00
40-49	422 (43.0)	50 (35.5)	0.86 (0.54-1.36)	0.76 (0.48-1.22)
50-59	241 (24.6)	34 (24.1)	1.02 (0.62-1.70)	0.83 (0.48-1.42)
≥60	126 (12.9)	27 (19.1)	1.66 (0.97-2.82)	1.21 (0.67-2.18)
Menopausal status				
Pre	610 (63.0)	74 (53.6)	1.00	1.00
Post	359 (37.0)	64 (46.4)	1.50 (0.93-2.42)	1.38 (0.85-2.25)
BMI, kg/m^2^ (mean(SD))	23 (2.9)	23 (3.2)	0.98 (0.92-1.04)	0.97 (0.91-1.03)
<25	753 (77.6)	107 (77.0)	1.00	1.00
≥25	217 (22.4)	32 (23.0)	0.85 (0.56-1.28)	0.79 (0.52-1.20)
Education				
Middle school	224 (22.9)	46 (32.6)	1.00	1.00
High school	402 (41.2)	56 (39.7)	0.68 (0.45-1.05)	0.69 (0.45-1.05)
College	351 (35.9)	39 (27.7)	0.58 (0.36-0.94)	0.58 (0.36-0.93)
Alcohol drinking				
<1/month	559 (57.5)	85 (60.7)	1.00	1.00
1-3/month	354 (36.4)	46 (32.9)	0.83 (0.57-1.21)	0.83 (0.57-1.22)
1+/week	59 (6.1)	9 (6.4)	1.12 (0.56-2.25)	1.09 (0.54-2.19)
Recruited sites				
SNU	511 (52.1)	73 (51.8)	1.00	1.00
AMC	469 (47.9)	68 (48.2)	0.92 (0.65-1.30)	0.89 (0.63-1.26)
TNM stage				
I	400 (40.8)	25 (17.7)	1.00	1.00
II	416 (42.5)	72 (51.1)	2.78 (1.74-4.44)	2.71 (1.69-4.35)
III	164 (16.7)	44 (31.2)	4.27 (2.57-7.11)	4.17 (2.50-6.96)
Tumor size				
<2 cm	526 (54.9)	42 (29.8)	1.00	1.00
≥2 cm	431 (44.9)	99 (70.2)	2.57 (1.74-3.79)	2.51 (1.70-3.72)
Tx	2 (0.2)			
Lymph node status				
Negative	577 (58.9)	60 (42.6)	1.00	1.00
Positive	390 (39.8)	78 (55.3)	1.47 (1.03-2.11)	1.46 (1.02-2.09)
Nx	13 (1.3)	3 (2.1)		
Hormone status				
ER + and/or PR+	698 (72.6)	74 (53.6)	1.00	1.00
ER–/PR–	264 (27.4)	64 (46.4)	2.06 (1.41-3.01)	2.06 (1.40-3.02)
Nuclear grade				
I-II	525 (55.2)	57 (41.0)	1.00	1.00
III	426 (44.8)	82 (59.0)	1.15 (0.78-1.69)	1.12 (0.75-1.65)

Abbreviation: AMC, Asan Medical Center; ER, estrogen receptor; PR, progesterone receptor; SNU, Seoul National University.

Data are presented as frequency (percentage), except for age and BMI in continuous variable, which are expressed as mean (SD).

^a^ Adjusted for age, TNM stage and hormone status and nuclear grade.

^b^ Adjusted for age, education, recruited site, TNM stage, hormone status and nuclear grade.

Table 2 presents the association between one-carbon metabolism related nutrients intake and disease progression in breast cancer patients. The mean intakes ± SD of vitamin B_2_, B_6_ and folate were 1.0 ± 0.4 mg, 1.5 ± 0.5 mg and 213.8 ± 99.3 mg respectively. Patients with a high (range, > median) intake of vitamin B_2_, B_6_, and folate had an increased HR for the recurrence compared to the patients with a low (range, < median) intake; however, the association was not statistically significant. When examining the association between the combined effects of vitamin B_2_, B_6_ and folate intake on the progression of breast cancer, the HR was 1.46 (95% CI, 0.87-2.43) for the high combined score group (range, 3) compared to the low combined score group (range, 0).

**Table 2:** Association between one-carbon metabolism related nutrients intake and disease progression in breast cancer patients

	**All (N = 980)**	**Events (N = 141)**	**Model 1**^**a**^	**Model 2**^**b**^
Vitamin B_2_ intake, mg				
Continuous (mean(SD))	1.0 (0.4)	1.0 (0.4)	1.21 (0.67-2.18)	1.29 (0.71-2.33)
1^st^ quartile (0.1-0.6)	192 (19.6)	29 (20.6)	1.00	1.00
2^nd^ quartile (0.7-0.8)	226 (23.1)	26 (18.4)	0.94 (0.54-1.61)	0.99 (0.58-1.73)
3^rd^ quartile (0.9-1.1)	295 (30.1)	44 (31.2)	1.20 (0.71-2.02)	1.28 (0.75-2.18)
4^th^ quartile (1.2-3.4)	267 (27.2)	42 (29.8)	1.40 (0.76-2.61)	1.58 (0.84-2.98)
*P*_trend_			0.21	0.12
Vitamin B_6_ intake, mg				
Continuous (mean(SD))	1.5 (0.5)	1.5 (0.6)	1.41 (0.90-2.20)	1.38 (0.88-2.17)
1^st^ quartile (0.1-1.0)	174 (17.8)	26 (18.4)	1.00	1.00
2^nd^ quartile (1.1-1.3)	262 (26.7)	39 (27.7)	1.27 (0.75-2.16)	1.28 (0.75-2.18)
3^rd^ quartile (1.4-1.7)	238 (24.3)	27 (19.2)	0.97 (0.72-1.80)	0.98 (0.53-1.83)
4^th^ quartile (1.8-4.8)	306 (31.2)	49 (34.8)	1.61 (0.82-3.16)	1.65 (0.84-3.24)
*P*_trend_			0.29	0.27
Folate intake, mg				
Continuous (mean(SD))	213.8 (99.3)	212.9 (98.3)	1.00 (0.99-1.00)	1.00 (0.99-1.00)
1^st^ quartile (9–147)	240 (24.5)	37 (26.2)	1.00	1.00
2^nd^ quartile (148–199)	248 (25.3)	32 (22.7)	0.82 (0.51-1.33)	0.82 (0.51-1.34)
3^rd^ quartile (200–256)	247 (25.2)	33 (23.4)	0.96 (0.58-1.60)	0.97 (0.58-1.63)
4^th^ quartile (257–970)	245 (25.0)	39 (27.7)	1.18 (0.68-2.03)	1.20 (0.69-2.07)
*P*_trend_			0.50	0.46
Combined score				
Low (0)	302 (30.8)	44 (31.2)	1.00	1.00
Medium (1–2)	289 (29.5)	35 (24.8)	0.92 (0.58-1.47)	0.93 (0.58-1.50)
High (3)	389 (39.7)	62 (44.0)	1.40 (0.84-2.33)	1.46 (0.87-2.43)
*P*_trend_			0.20	0.15

Data are presented as frequency (percentage), except for continuous variable, which are expressed as mean (SD).

^a^ Adjusted for age, TNM stage, hormone status, nuclear grade and total calorie.

^b^ Adjusted for age, education, recruited site, TNM stage, hormone status, nuclear grade and total calorie.

Table 3 presentes the association between the median of one-carbon metabolism related nutrients intake and disease progression in breast cancer patients stratified by clinicopathological characteristics. No differential association was found between the intake levels of vitamin B_2_, B_6_, and folate and disease progression stratified by TNM stage, lymph-node status, or nuclear grade. However, in ER–/PR– breast cancers, a high intake of vitamin B_2_ and folate statistically elevated the HR of breast cancer progression compared to a low intake (HR = 2.28; 95% CI, 1.20-4.35, HR = 1.84; 95% CI, 1.02-3.32, respectively). Low *versus* high (reference = low) intake of vitamin B_6_ showed an increased HR for breast cancer progression; however, the association was not significant (P_interaction_ = 0.04, HR = 1.86; 95% CI, 0.97-3.56).

**Table 3:** Association between median of one-carbon metabolism related nutrients and disease progression in breast cancer patients stratified by clinicopathological characteristics

		**Below median**		**Above median**		
		**No. of total**	**No. of events**	**No. of total**	**No. of events**	**HR (95%CI)**^**a**^
	TNM stage					
Vitamin B_2_, mg	I-II	350	39	466	58	1.40 (0.85-2.30)
	III	68	16	96	28	1.13 (0.52-2.43)
Vitamin B_6_, mg	I-II	362	46	454	51	1.02 (0.61-1.73)
	III	74	19	90	25	1.05 (0.49-2.27)
Folate, mg	I-II	397	48	419	49	1.10 (0.69-1.75)
	III	91	21	73	23	1.52 (0.77-3.01)
	Lymph-node status					
Vitamin B_2_, mg	Negative	242	25	335	35	1.28 (0.67-2.42)
	Positive	172	29	218	49	1.40 (0.81-2.44)
Vitamin B_6_, mg	Negative	251	28	326	32	1.22 (0.63-2.34)
	Positive	181	36	209	42	0.88 (0.49-1.56)
Folate, mg	Negative	284	30	293	30	1.23 (0.68-2.24)
	Positive	199	38	191	40	1.12 (0.67-1.87)
	Hormone status					
Vitamin B_2_, mg	ER + and/or PR+	300	34	398	40	0.91 (0.52-1.59)
	ER–/PR–	111	21	153	43	2.28 (1.20-4.35)
Vitamin B_6_, mg	ER + and/or PR+	305	39	393	35	0.57 (0.32-1.03)
	ER–/PR–	125	26	139	38	1.86 (0.97-3.56)
Folate, mg	ER + and/or PR+	333	39	365	35	0.82 (0.49-1.40)
	ER–/PR–	148	30	116	34	1.84 (1.02-3.32)
	Nuclear grade					
Vitamin B_2_, mg	I-II	223	23	302	34	1.22 (0.64-2.34)
	III	185	32	241	50	1.43 (0.82-2.49)
Vitamin B_6_, mg	I-II	226	29	299	28	0.56 (0.28-1.11)
	III	200	36	226	46	1.28 (0.73-2.24)
Folate, mg	I-II	250	31	275	26	0.69 (0.38-1.27)
	III	224	38	202	44	1.59 (0.95-2.66)

Abbreviation: ER, estrogen receptor; PR, progesterone receptor.

^a^ Adjusted for age, education, recruited site, TNM stage, hormone status, nuclear grade and total calorie.

Furthermore, we evaluated the association between the combined scores of one-carbon metabolism related nutrients intake and disease progression in breast cancer patients stratified by hormone status (Table 4). The positive association between ER/PR status was profound when the nutrient intakes were categorized by combined score (P_interaction_ = 0.018). In ER–/PR– cancers, the high combined score (range, 3) group was associated with a significantly poor DFS compared to those belonging to the low score (range, 0) group (HR = 3.84; 95% CI, 1.70-8.71).

**Table 4:** Association between combined score of one-carbon metabolism related nutrients intake and disease progression in breast cancer patients stratified by selected characteristics

	**Combined score**	**No. of total**	**No. of events**	**HR (95%CI)**^**a**^
Hormone status^b^				
ER + and/or PR+	Low (0)	211	29	1.00
	Medium (1–2)	202	16	0.54 (0.29-1.02)
	High (3)	285	29	0.72 (0.37-1.43)
	*P*_trend_			0.29
ER–/PR–	Low (0)	87	15	1.00
	Medium (1–2)	82	19	2.02 (0.96-4.27)
	High (3)	95	30	3.84 (1.70-8.71)
	*P*_trend_			0.001

Abbreviation: ER, estrogen receptor; PR, progesterone receptor.

^a^ Adjusted for age, education, recruited site, TNM stage, hormone status, nuclear grade and total calorie.

^b^*P* for interaction = 0.018.

## Discussion

There was no association between one-carbon metabolism related nutrients and disease free survival of breast cancer overall, while some effects were observed when stratifying by breast cancer subtypes. To the best of our knowledge, this study is the first to systematically investigate the association between the combined effects of prediagnostic intake of nutrients related to the one-carbon metabolism pathway and the clinicopathological characteristics of breast cancer.

In our study, the range for the intake of vitamin B_2_, B_6_ and folate were 0.1-3.4 mg, 0.1-4.8 mg and 9–970 mg, respectively. These levels of vitamin B_6_ and folate were less than the tolerable upper intake level of Dietary Reference Intakes for Koreans (KDRIs). The tolerable upper intake of vitamin B_2_ level was not determinable by the KDRIs. In the KDRIs, the tolerable upper intake level of vitamin B_6_ and folate were 100 mg and 1000 mg. The levels of dietary intake of vitamin B_2_, B_6_, and folate in breast cancer patients were hardly reported. Even though there were many previous studies on the association between vitamin B_2_, B_6_ and folate and breast cancer risk, they reported the tertiles or quartile levels in both the cases and the controls. Thus, it is hard to compare the nutrients intake level of patients’ with other studies that used different designs.

In observational studies, the results for the relationship between micronutrient intakes and all-cause mortality were inconsistent among breast cancer patients [21]. Only one study investigated one-carbon metabolism related nutrients intake and all-cause of mortality in women with breast cancer, and showed null results for vitamin B_2_, B_6_ and folate [17]. Few studies have investigated the association between folate intake and breast cancer survival. McEligot et al. studied 516 postmenopausal women diagnosed with breast cancer for 6.6 years (median follow-up) and showed that women in the highest tertile for dietary folate intake had an HR of 0.34 (95% CI, 0.18-0.67) regarding all-cause mortality [22]. In addition, in the Swedish mammography cohort, dietary folate intake was inversely associated with overall mortality (HR = 0.79; 95% CI, 0.66-0.96) [16]. However, in the Iowa Women’s Health Study, Sellers et al. reported that among 177 breast cancer patients, folate intake had no association with breast cancer prognosis [23]. Moreover, in the Nurses’ Health Study, Holmes et al. provided additional support for no association of breast cancer prognosis with vitamin B_2_, B_6_ and folate [18]. In addition, Rossi et al*.* measured the folate levels in plasma from 1024 breast cancer patients in Australia and reported that plasma folate was not significantly associated with breast cancer survival [24]. No other prior studies have reported the relationship between one-carbon metabolism related factors and their combined intake effects on the prognosis of breast cancer. The lack of consensus in the results of previous studies could be explained by the variation in study methodologies, micronutrient source, and total micronutrient level. Thus, it is difficult to determine how the micronutrient intake distribution reported in one study compares to other studies [25].

We observed that the combined intake effects of vitamin B_2_, B_6_, and folate were associated with breast cancer progression in patients depending on their ER/PR status. No previous studies examining the combined intake effects of one-carbon metabolism related nutrients intake and hormone specific breast cancer have been identified. In the Swedish Mammography Cohort (SMC), only folate intake, which is one of the one-carbon metabolism related nutrients studies, was assessed for association with breast cancer progression [20]. Although Harris et al. have reported that dietary folate intake has shown protective effects on breast cancer-specific mortality in ER-negative tumors, our results do not support this effect. It is difficult directly compare our study’s results to theirs since the distribution of the hormone receptors in the study subjects was different (ER-negative breast cancer, less than 20% in SMC; 39.2% in the present study). For breast cancer risk, few studies have evaluated the relationship between hormone status and folate intake. A higher folate intake was associated with a lower risk of ER-negative breast cancer in the Nurses’ Health Study [26], and in the VITamins And Lifestyle (VITAL) cohort [27]. In SMC, a high folate intake was related to a decreased risk of ER+/PR– breast cancer [28]. Otherwise, few cohort studies have reported largely null results, with no findings for associations between folate intake and ER+, ER–, PR + or PR– breast cancers [26,27,29,30]. From various laboratory studies, their results were inconsistent with our study, which examined whether DNA methylation of the ER CpG island may play a role in suppressing ER gene expression in ER-negative breast cancer cells [31,32]. A greater supply of methyl through a high intake of one-carbon metabolism related nutrients may induce the suppression of gene expression in patients with ER–/PR– breast cancers. In addition, a high intake of one-carbon metabolism related nutrients may have a stronger effect on ER–/PR– breast cancer progression than the other types of breast cancers since ER–/PR– breast cancers are less responsive to hormone therapies. Further studies are needed to elucidate the possible association between one-carbon metabolism related nutrients and the hormone receptors in breast cancer patients.

The results of our study were contrary to the hypothesis based on previous studies that a higher intake of B vitamins would have a protective effect on breast cancer survival in population based studies. One prospective cohort study suggested the association of major energy sources with breast cancer survival may be U-shaped rather than linear [33]. As far as this idea, the association has not yet been proven; however, a midrange intake of vitamins is associated with the most favorable outcomes, and extremes are associated with less favorable outcomes.

This study has some limitations. First, there were likely errors in our estimate of dietary habits. Patients were asked about their dietary intake for the year preceding the diagnosis using the FFQ. Due to this, measurement errors likely occurred because of poor recall despite the validity and reproducibility evidence of the questionnaire. The FFQ correlations were lower than that reported in western countries which were between 0.5-0.7 [34]. In Asia, the median of the correlation coefficients for the FFQ has ranged from 0.3-0.5 in Japan [35,36] and Korea [37,38], and a lower FFQ correlation may have been caused by the dining etiquette and cultural foods of Korea [20]. Though the correlation coefficients were low, to date, FFQ is the only method in which long-term usual dietary intake of an individual can be easily obtained with a single measurement [39]. Second, intake of supplements was not available for the calculations. However, the intake of supplements use can improve the dietary quality for certain micronutrients [25]. Lastly, we could not evaluate the association between one-carbon metabolism related nutrients and breast cancer specific mortality because the data for the cause of death were not available. Thus, the results must be interpreted cautiously and need to be confirmed by a study that investigates the association of one-carbon metabolism related nutrients with breast cancer specific survival.

Nevertheless, our study has strengths. It is the first study to evaluate the association between the combined intake effects of vitamin B_2_, B_6_ and folate and hormone specific breast cancer survival.

## Conclusion

In summary, one-carbon metabolism related nutrients are associated with disease free survival depending on the ER/PR status among breast cancer patients. However, because of the small population in these subgroup analyses, these results should be interpreted with caution. Future studies examining the pathways of one-carbon metabolism related nutrients in certain breast cancer types must account for the direct or indirect roles of these nutrients.

## Abbreviations

AMC, Asan Medical Center; CI, Confidence interval; DFS, Disease free survival; DRs, Diet records; ER, Estrogen receptor; FFQ, Food frequency questionnaire; HR, Hazard ratio; KDRIs, Dietary Reference Intakes for Koreans; PR, Progesterone receptor; SD, Standard deviation; SNU, Seoul National University; SMC, Swedish Mammography Cohort.

## Competing interests

The authors declare that they have no competing interests.

## Author’s contributions

DK, DYN, and SHA were PIs for each of the participating cooperative groups of the Seoul Breast Cancer Study (SeBCS). YL, SAL, JYC, SKP, KYY and DK were involved in conception and design of the study and participated in the discussion and interpretation of the results. YL carried out data analysis and writing of the manuscript. MS, HS and SJ contributed to statistical analyses and helped to draft the manuscript. All authors have read and approved the final manuscript.
